# Reduction of left ventricular ejection fraction after 12-month follow-up in hemodialysis patients

**DOI:** 10.15171/jrip.2016.02

**Published:** 2016-02-24

**Authors:** Ali Momeni, Arsalan Khaledi, Katayoun Hasanzadeh

**Affiliations:** ^1^Department of Internal Medicine, Shahrekord University of Medical Sciences, Shahrekord, Iran

**Keywords:** Echocardiography, Hemodialysis, Left ventricular ejection fraction

## Abstract

**Introduction:** Cardiovascular disease is the most common cause of morbidity and mortality in hemodialysis patients.

**Objectives:** The aim of this study was to detect echocardiographic abnormality in the beginning and after 12-month follow-up in the hemodialysis patients.

**Patients and Methods:** In a cross-sectional study, 60 hemodialysis patients older than 18 years and the dialysis duration longer than three months were enrolled. At the beginning of the study, echocardiography was done and after 12 months was repeated in all of the patients by the same cardiologist. At the end of the study, data were analyzed using SPSS software (version 19).

**Results:** From the total of cases 37 were male and 23 female. At the beginning of the study, mitral regurgitation, tricuspid regurgitation and aortic insufficiency were found in 54, 47 and 11 patients respectively. After 12 months left ventricular ejection fraction (LVEF) decreased significantly, however there was no significant difference between other echocardiographic findings at the beginning and after 12 months.

**Conclusion:** Decrease in LVEF over time in hemodialysis patients may be due to negative effect of uremia on cardiac function, so it seems that periodical cardiac evaluation of these patients is essential and beneficial.

**Implication for health policy/practice/research/medical education:**

In a cross-sectional study, 60 hemodialysis patients older than 18 years and the dialysis duration longer than three months were enrolled. Echocardiography was done and was repeated in all of the patients. From the total of cases 37 were male and 23 female. After 12 months left ventricular ejection fraction (LVEF) decreased significantly, however there was no significant difference between other echocardiographic findings at the beginning and after 12 months. Decrease in LVEF over time in hemodialysis patients may be due to negative effect of uremia on cardiac function, so it seems that periodical cardiac evaluation of these patients is essential and beneficial.

## Introduction

Cardiovascular disease is responsible for about 60% of mortality rate in hemodialysis patients that is 30 times more prevalent than in general population (1-2). Some predisposing factors to sudden death in these patients include left ventricular hypertrophy (LVH), electrolyte shifts in hemodialysis session and abnormalities in myocardial ultrastructure and function (3). Different cardiovascular diseases, such as LVH, coronary artery diseases, congestive heart failure, and hypertension are commonly seen in these patients (4,5). In addition, calcification of cardiac valves is common and can cause valvular and annular thickening which in turn could lead to valvular stenosis or regurgitation (6). Some predisposing factors of cardiac disorders in dialysis patients are secondary hyperparathyroidism, long-term hypertension, anemia, large inter-dialytic fluid gains, uremic internal milieu, and presence of vascular grafts and arteriovenous fistulae (7,8). Left ventricular dysfunction and LVH are poor prognostic factors and increase mortality in hemodialysis patients (9,10). National Kidney Foundation recommend baseline and routine echocardiographic follow-up for end-stage renal disease (ESRD) patients (11). In chronic kidney disease patients, there is a stepwise increase in LVH and reduction in left ventricular ejection fraction (LVEF) with progression of renal failure from stage 3 to stage 5 (12). With initiation of dialysis left ventricular mass index may be decreased compared to prior to dialysis probably due to relief of venous congestion (13).

## Objectives

There is little research on change of echocardiographic findings during a long time follow-up, so the aim of the present study is to evaluate echocardiographic changes during 12 months in hemodialysis patients.

## Patients and Methods

### Study population

In this cross-sectional study, 60 hemodialysis patients older than 18 years and the dialysis duration longer than three months were enrolled.

### Data collection

At the beginning of the study echocardiography was done in all of the patients and repeated by the same cardiologist after 12 months. Echocardiography was performed after the dialysis session using Vivid S6 Machine (USA). The patients were on hemodialysis, by Fresenius digital machine (4008B, Germany) and Gambro digital machine (AK95 and AK96, Sweden), 2 to 3 times per week. Dialysis duration was 4 to 4.5 hours per session with blood flow (QB) of 250 to 350 ml/min and dialysate flow (QD) of 500 ml/min. Ultrafiltration was based on the patient’s condition (about 1 to 3 liter each time); however, at the end of dialysis session the patients were in their dry weight.

The used buffer was bicarbonate powder and the type of filters were intermediate and high efficient polysulfide membrane of R6 or R7 (SOHA, Fresenius Company). For measurement of urea reduction ratio (URR) and KT/V in the patients, serum BUN and Cr were checked in the same laboratory before and after hemodialysis session. To undertake echocardiography, an informed consent was confidentially filled out by the patients. In the echocardiography in this study, LVEF, LVH, pulmonary artery pressure, and valvular disorders were evaluated.

### Exclusion criteria

Exclusion criteria were antiarrhythmic drugs consumption, history of cardiac disease such as arrhythmia, heart block or congestive heart failure.

### Ethical issues

1) The research followed the tenets of the Declaration of Helsinki; 2) informed consent was obtained, and they were free to leave the study at any time and 3) the research was approved by the ethical committee of Shahrekord University of Medical Sciences.

### Statistical analysis

At the end of this study, data were analyzed using SPSS software (version 19). *P* value less than 0.05 was considered as significant. Pearson correlation coefficient, two-independent samples *t* test and analysis of variance (ANOVA) were used for statistical analysis.

## Results

From the total of 60 patients, 37 were male and 23 female. The causes of renal failure were diabetes mellitus, hypertension, hereditary kidney disease, chronic glomerulonephritis, and unknown in 26, 15, 8, 5 and 6 patients, respectively. Mean age of the patients was 56.15±14.6 years. Mean body mass indexes (post dialysis) and duration of dialysis were 21.77±3.6 kg/m^2^ and 4.25±3.24 years respectively (Table 1). Mean URR in men and women was 68.14±9.34 and 68.22±3.88 respectively; however, KT/V was 1.45±0.17 and 1.49±0.16 in men and women, respectively. In the beginning of the study LVH was seen in 23 (38.3%) of the patients based on septal thickness greater than 10 mm, and ST-T change in14 patients (23.3%). In echocardiography, mitral regurgitation (MR), tricuspid regurgitation (TR) and aortic insufficiency (AI) were found in 54, 47 and 11 patients, respectively. In the second echocardiography (after 12 months), in 38 patients (63.3%) LVEF did not change, in 18 patients (30%) decreased and in 4 patients (6.7%) increased, so LVEF decreased significantly compared to the beginning of the study by paired *t* test (*P*=0.007). At the end of the study, LVEF was not correlated with MR (*P*=0.14), AI (*P*=0.11) or TR (*P*=0.39). However, there was no significant difference among other abovementioned echocardiographic findings at the beginning of the study and after 12 months in the studied patients (Table 2).

**Table 1:** Demographic and laboratory findings of patients

**Characteristic**	**Age (year)**	**Body weight (kg)**	**BMI (kg/m** ^2^ **)**	**Duration of dialysis (year)**	**Dialysis/week**
Value (Mean±SD)	56.15±14.6	62.13±14.54	21.77±3.6	4.25±3.24	2.9±0.3

**Table 2:** Echocardiographic findings of the patients before and after the study

**Echocardiographic variable**	**Value**	***P***
EF1 (%)	51.92±4.60	0.007
EF2 (%)	50.42±5.30	
PAP1 (mm Hg)	33.83±13.25	0.1
PAP2 (mm Hg)	31.58±12.63	
Septal thickness 1(mm)	13.23±1.60	0.08
Septal thickness 2 (mm)	12.97±1.50	

Abbreviations: EF‏, Ejection fraction; PAP, Pulmonary arterial pressure.

## Discussion

Chronic effect of hemodialysis on left ventricular (LV) function has not been well understood. Our results showed that LVEF of the patients decreased significantly after 12 months despite the acceptable adequacy of hemodialysis. Valvular disorders and LVH also were common even in the patients without obvious cardiac disease but there was no difference between these findings at the beginning and end of this study.

In hemodialysis patients, due to chronic hypertension, volume overload, and high cardiac load (because of presence of an AVF and chronic anemia), LV diastolic and systolic dysfunction may occur and ejection fraction eventually decreases (14). There are a few studies about the echocardiographic changes over time. For example, Duran et al in 22 newly diagnosed cases of ESRD showed that LV systolic functions, LV diameters, LV mass index, left atrium size, and RV diastolic functions did not statistically change after long-term hemodialysis treatment (15). In our study decrease in LV function over time in the patients may be due to negative effect of uremia on heart muscle fibers (16). In hemodialysis patients, chronic volume overload, LVH, chronic anemia and electrolyte imbalance are predisposing factors of cardiomyopathy and decrease in LVEF (17). Discrepancy between the results of Duran et al and our study is probably due to difference in the scale of the study. The majority of other researches focused on the changes during the dialysis session. In Wu et al study on hemodialysis patients, where the evaluation was done by three dimensional echocardiography, there was no significant change in LVEF before and after hemodialysis session (18). LVH in hemodialysis patients is common and is caused due to chronic hypertension, anemia and other factors such as hyperparathyroidism. Prevalence of LVH in hemodialysis patients was reported 42% to 75% based on duration of renal failure (19). In our study LVH was found in 38.3% of the patients and did not change after 12 months. LVH and diastolic dysfunction are more common in hemodialysis patients with diabetes mellitus. Accordingly, Hung et al in a study on 101 hemodialysis patients with normal systolic function found that diabetes mellitus patients had advanced LV diastolic dysfunction compared to non-diabetic patients (20). In addition to LVH, diastolic dysfunction may be developed due to regional wall motion abnormalities (RWMA). For example Assa et al showed that hemodialysis could induce RWMA in a significant proportion of patients, and these abnormalities may lead to increased mortality rate (21).

Also, our results showed that there were valvular disorders in a considerable number of the patients. In some other studies, similar to our results, valvular disorders in hemodialysis patients were common. Risk factors of valvular disorders in hemodialysis patients are calcification of heart valves and other factors such as anemia, LVH and hypertension (22-24).

## Conclusion

Our results showed that LVEF decreased over time in hemodialysis patients, probably due to negative effect of renal failure and hemodialysis on cardiac function. So, it seems that annually cardiac evaluation and follow-up of these patients are essential and beneficial.

## Limitations of the study

The study had some limitations such as small sample size and short duration of follow-up, thus we recommend to conduct of similar studies as multi-centric with longer duration of follow-up.

## Authors’ contribution

AM; study design, preparation of manuscript and final revision. AK; study design, echocardiography and final revision. KH; data gathering and data interpretation.

## Conflicts of interest

The authors declared no competing interests.

## Ethical considerations

Ethical issues (including plagiarism, data fabrication, double publication) have been completely observed by the authors.

## Funding/Support

This research is part of an internal medicine residential thesis dissertation that was financially supported by grant No. 904 from Shahrekord University of Medical Sciences. The authors of this research intend to thank all who support us both technically and financially.
